# Chromosome-level reference genome for the common wall gecko (*Tarentola mauritanica*) enables comparative and functional studies in geckos

**DOI:** 10.1093/g3journal/jkag072

**Published:** 2026-03-31

**Authors:** Gabriel Mochales-Riaño, Valéria Marques, Miguel A Carretero, Catarina Rato

**Affiliations:** New York University Abu Dhabi, Department of Biology, Abu Dhabi, United Arab Emirates; Institute of Evolutionary Biology (IBE), CSIC-Universitat Pompeu Fabra, Barcelona 08003, Spain; CIBIO, Centro de Investigação em Biodiversidade e Recursos Genéticos, InBIO Laboratório Associado, Campus de Vairão, Universidade do Porto, Vairão 4485-661, Portugal; BIOPOLIS Program in Genomics, Biodiversity and Land Planning, CIBIO, Campus de Vairão, Vairão 4485-661, Portugal; Departamento de Biologia, Faculdade de Ciências, Universidade do Porto, Porto 4099-002, Portugal; APH, Associação Portuguesa de Herpetologia, Porto 4099-002, Portugal

**Keywords:** genomics, gekkota, aquaporins, genome assembly

## Abstract

Reptiles exhibit remarkable ecological and physiological diversity, yet genomic resources for this group remain relatively scarce, limiting research on adaptation, evolution, and invasion biology. The common wall gecko, *Tarentola mauritanica* (Linnaeus, 1758), is a Mediterranean species complex known for its urban behavior, wide dispersal capacity, and exceptional resistance to dehydration, making it an ideal candidate for genomic studies of ecological adaptation. Here, we present the first high-quality, chromosome-level reference genome for the genus *Tarentola*. Using a combination of PacBio HiFi and Hi-C sequencing data, we assembled a 2.1 Gb reference genome (N50 = 135.8 Mb), of which 96.7% of the genome is contained in 21 pseudochromosomes. Comparative chromosomal analyses revealed largely conserved synteny, however, some fissions and fusions were observed, highlighting lineage-specific karyotype evolution. Analyses of aquaporin genes revealed a duplication of *AQP5* in *T. mauritanica*, suggesting a potential role in water regulation. PSMC-based demographic reconstruction indicated population expansion prior to the Last Interglacial (∼150 to 120 Kya) and contraction during the Last Glacial Maximum (∼20 Kya), consistent with Mediterranean climatic fluctuations. This genome represents the first reference for the genus *Tarentola* and provides a comprehensive genomic resource to investigate ecological adaptation, gene family evolution, invasion biology, and conservation genomics. Moreover, this assembly will facilitate comparative genomics across reptiles and support functional and evolutionary studies aimed at linking genome structure to ecological and phenotypic diversity.

## Introduction

Genomic resources are essential tools for understanding biodiversity, as they are the basis for addressing questions ranging from phylogenomics to functional biology (Theissinger et al. 2023). High-quality reference genomes, in particular, enable the study of evolutionary processes at multiple scales, from reconstructing deep divergences to identifying fine-scale genetic variation within populations (Rhie et al. 2021; Theissinger et al. 2023). Moreover, reference genomes provide a critical framework for applied fields such as conservation genomics, where they can inform about the management of endangered species, and invasion genomics, where they help uncover the genetic basis of colonization success, rapid adaptation, and range expansion (McCartney et al. 2019; Heuertz et al. 2023). In reptiles, genomic resources remain relatively scarce compared to other vertebrate groups despite their ecological importance, unique physiological adaptations, and biomedical relevance (although it has been increasing, see Andrade et al. 2019; Burriel-Carranza et al. 2024; Margres et al. 2021; Mochales-Riaño et al. 2025; Schield et al. 2019; Talavera et al. 2025, 2026, among others).

Beyond their role in evolutionary and comparative genomics, reference genomes open the door to investigating specific biological systems such as coloration, domestication, or venom (Andrade et al. 2019; Schield et al. 2019; Frantz et al. 2020; Suryamohan et al. 2020; Margres et al. 2021). Another interesting feature within reptile genomics lies on the study of gene families, such as aquaporins (*AQP*), a superfamily associated with water balance and osmoregulation, processes critical for survival in arid and fluctuating environments, and, hence, of paramount importance to infer potential responses to climate change. These small membrane intrinsic proteins (24 to 36 kDa) form selective pores that facilitate the movement of water and other small solutes across cellular membranes (Kruse et al. 2006; Abascal et al. 2014). Their diversification in vertebrates reflects adaptation to distinct ecological niches and physiological demands, and reptiles offer an ideal system for studying their evolution (Finn et al. 2014; Araya-Donoso et al. 2021; Wollenberg et al. 2022). Among aquaporins, AQP2 and AQP5 are of particular interest: AQP2 plays a central role in water reabsorption in the kidney and is tightly regulated by vasopressin, while AQP5 contributes to fluid secretion in exocrine glands (Burghardt et al. 2006; Wilson et al. 2013). Comparative analyses of these genes in reptiles could shed light on how different lineages have adapted to environments with contrasting water availability, including deserts and Mediterranean ecosystems.

The common wall gecko, *Tarentola mauritanica* (Linnaeus 1758), represents an excellent candidate to be studied from a genomic perspective. This mid-sized gecko constitutes a species complex comprising six putatively distinct taxa (Rato et al. 2016), with a native distribution restricted to the western coastal Mediterranean Basin (Vogrin et al. 2017). Owing to its synanthropic behavior and capacity for long-distance dispersal (Carranza et al. 2000), the species has expanded beyond its native range across several Mediterranean, tropical and subtropical regions of the globe, where it has found novel environmental conditions (e.g. Rato et al. 2015; Díaz-Fernández et al. 2019; Ortiz-Medina et al. 2019; Deso et al. 2020, Rato et al. 2023a , 2023b). A recent Ecological Niche Modeling study demonstrates that the realized niche of introduced populations differs significantly from that of their native counterparts (Rato et al. 2024), underscoring the species’ capacity to adapt to novel ecosystems, an ability that is critical for successful colonization. Moreover, the study suggests that over the next six decades, the geographic distribution of this gecko will likely expand in response to global climate change. Compared with other sympatric lizards of similar size, *T. mauritanica* exhibits much greater resistance to dehydration (approximately 10 times higher) (García-Muñoz and Carretero 2013; Osojnik et al. 2013; Le Galliard et al. 2021), with substantial intraspecific plasticity in this trait across populations within the invasive lineage of the complex (Rato and Carretero 2015). This physiological advantage enables the common wall gecko to endure prolonged periods without water, facilitating its survival during transmarine dispersal events.

Hence, despite being one of the most conspicuous gecko species in human-modified environments, no reference genome is currently available for this taxon or even for the genus *Tarentola*, which comprises 33 described species (Uetz 2025). The assembly of the first reference genome for *T. mauritanica*, provides not only a valuable resource for understanding the biology and adaptability of this invasive species, but also the basis for comparative studies across the genus and for investigating the evolution of key gene families, such as those involved in osmoregulatory processes.

## Materials and methods

### Sampling

In May 2024, 2 adult specimens (1 female and 1 male) of *Tarentola mauritanica* were captured with a noose in Vialonga, Portugal (38.875784N, 9.085495W). Stomach and liver tissues were collected from the female individual to obtain high-molecular-weight (HMW) genomic DNA (gDNA), and muscle tissue to build the Hi-C library. All samples were stored at —80 °C immediately after being harvested. From the male individual, 4 different tissues (kidney, gonads, brain, and liver) were collected, and stored at—80 °C until RNA extraction. Although sex in *T. mauritanica* has been widely reported to follow a temperature-dependent sex determination mechanism (Marques et al. 2023), we selected a female individual for genome assembly as a precautionary measure, given that closely related taxa with genetic sex determination exhibit a ZZ/ZW system in which females are the heterogametic sex (Gamble et al. 2015).

### DNA extraction, library preparation, and sequencing

gDNA was extracted from the stomach and liver tissues of the female individual, using the MagAttract HMW Kit (Qiagen), and following the manufacturer's protocols. Then, from the stomach's HMW, a single SMRT Cell run on the PacBio Revio system was sequenced, aiming for a 30 × of coverage. Main steps in Hi-C technology include DNA cross-linking, digestion by a restriction enzyme, end-repairing, circularization, and DNA purification (Lieberman-Aiden et al. 2009), using the female's muscle tissue. The Hi-C library was paired-end and sequenced on an Illumina Novaseq PE150 (2 × 150 bp), following the manufacturer's protocol for dual indexing and aiming for a coverage of ∼60×. Finally, short-read whole-genome sequence data were obtained using the female's liver tissue with the Illumina Novaseq X PE150, aiming for 30 × depth of coverage. Hi-C library and sequencing services were performed by Biomarker Technologies GmbH (Münster, Germany).

### RNA extraction, library preparation, and sequencing

RNA was isolated using the TGuide Smart Magnetic Tissue DNA Kit (TIANGEN BIOTECH) (Beijing, China) from the kidney, gonads, brain, and liver harvested from the male specimen. RNA libraries were prepared using a 12GB stranded poly-A mRNA enrichment, using the Illumina Novaseq PE150 as the sequencing strategy. RNA extraction, library preparation, and sequencing were performed by Biomarker Technologies GmbH (Münster, Germany).

### Genome assembly and scaffolding

Quality control of HiFi, Hi-C, and Illumina reads was performed using FastQC v.0.12.1 (Andrews 2010) and adapters were removed with cutadapt v.4.9 (Martin 2011) and fastplong v.0.3.0 (Chen 2023). Then, the reads were assembled following the VGP assembly pipeline v.2.0 (Rhie et al. 2021). PacBio HiFi reads were assembled into contigs using Hifiasm v.0.21.0 (Cheng et al. 2021), producing primary and alternate assemblies. Purge_dups (Guan et al. 2020) was used to remove haplotypic duplicates from the primary assembly and add them to the alternate assembly. Then, the resulting assembly was scaffolded using the Hi-C data with the software SALSA2 v.1 (Ghurye et al. 2019), with default parameters. Following the VGP assembly pipeline (Rhie et al. 2021), the manual curation was performed with Pretext v.0.2.5. Breaks were not manually created and contigs were only joined on gaps previously identified by the SALSA2 software. Once the assembly was generated, the mitochondrial genome was obtained with GetOrganelle v.1.7.7.0 (Jin et al. 2020), using the available mitochondrial genome of the Atlas day gecko, *Quedenfeldtia trachyblepharus* from Lyra et al., (2017) to seed the assembly.

### Genome assembly quality evaluation

Quality assessment and general metrics for our final assembly were estimated with both QUAST v.5.1.0 (Gurevich et al. 2013) and gfastats v.1.3.8 (Formenti et al. 2022). Possible contaminations were evaluated with BlobToolKit v.4.4.0 (Challis et al. 2020) using the NCBI taxdump database. MitoFinder v.1.4.2 (Allio et al. 2020) was also used to confirm that the mitochondrial genome was absent in the assembled nuclear reference genome. Finally, completeness of the genome assembly was assessed with BUSCO v.5.3.0 (Simão et al. 2015) against the sauropsida odb10 database (n = 7,480).

### RNA-seq and genome annotation

Before annotating the genes, repetitive elements in the newly assembled reference genome were characterized using a combined *de novo* and homology-based approach. First, a species-specific repeat library was generated using RepeatModeler v.2.0.3 (Flynn et al. 2020), which performs *de novo* identification and classification of repeat families directly from the genome assembly. Second, we performed an initial homology-based annotation using RepeatMasker v.4.1.3 (Tempel 2012) with the curated Repbase Tetrapoda library (Bao et al. 2015) to identify known vertebrate transposable elements and other repetitive sequences. Third, we conducted 3 iterative rounds of RepeatMasker using the custom repeat library produced by RepeatModeler. In these runs, both the Repbase Tetrapoda library and the species-specific *de novo* library were provided to RepeatMasker. After each round, the newly classified elements were incorporated into the custom library to improve repeat classification and reduce the proportion of sequences labeled as “unknown.” This iterative strategy aimed to maximize the annotation of repeat families while minimizing unclassified repetitive content. Finally, the genome assembly was soft-masked based on the final RepeatMasker output and used for downstream gene annotation. Then, adapters were trimmed and fastq reads were filtered for the RNA-seq data using fastp v.0.23.3 (Chen et al. 2018a). The obtained RNA-seq data were mapped to the new reference genome of *T. mauritanica* using Hisat2 v.2.2.1 (Kim et al. 2019). Then, in order to annotate protein-coding genes, GeMoMa v.1.9 (Keilwagen et al. 2019) was implemented, combining both the RNA-seq data generated in this study as well as the annotation produced for the Leopard gecko, *Eublepharis macularius* (Pinto et al. 2023). The RNA-seq data used for annotation were obtained from a male individual, whereas the reference genome was assembled from a female. Given that *T. mauritanica* has been reported to exhibit temperature-dependent sex determination (see Marques et al., 2023), we do not expect substantial sex-linked genomic differentiation. Nevertheless, we acknowledge that strictly male-specific transcripts, if present, may not be fully represented in the assembled genome.

### Chromosome-level analyses

The study of chromosomal synteny was explored between several lizard species (most of them geckonids) with available reference genomes, together with our newly assembled reference genome: the genomes of the Brown anolis (*Anolis sagrei*), the Leopard gecko (*Eublepharis macularius*), the Bynoe's gecko (*Heteronotia bynoei*), and the Emirati leaf-toed gecko (*Asaccus caudivolvulus*) were used, together with our newly assembled reference genome for the Common wall gecko (*Tarentola mauritanica*) using MCscan v.1.4.23 (Tang et al. 2008). Protein sequences from each species were extracted using AGAT v.1.2.1 (Dainat et al. 2023) and were pairwise aligned with LAST (Kiełbasa et al. 2011), implemented in the JCVI Python module (Tang, et al. 2017). Chromosome pairs in reverse orientation were not flipped, as a high number of genes were lost during the process.

### Local synteny analyses

To explore the Aquaporin genomic organization within the genes with gene copy number variation (i.e. *AQP5*), the associated region was extracted from each of the reference genomes used for the macrosynteny analyses. For each species, we retrieved the genomic interval spanning the annotated AQP5 locus plus ± 50 kb flanking regions to ensure inclusion of neighboring genes. In *Heteronotia*, neither the *AQP2* nor *AQP5* genes were annotated in the available gene set. To identify these loci, we performed BLAST searches using the *T. mauritanica AQP5* gene as a query against *Heteronotia*. Putative hits were manually inspected to confirm exon structure and coding potential. The *AQP2* gene was subsequently identified based on sequence similarity and conserved syntenic position. Once the genomic regions were selected for each species, those regions were aligned using Clustal Omega 1.2.3, implemented Geneious Prime 2025.1.2. Each species was annotated within the MSA using its own annotation as a reference in Geneious Prime 2025.1.2. Results were plotted using the gggenomes package (Hackl et al. 2024) from R (R Core Team 2021).

### Protein phylogeny

Phylogenetic inference was implemented in order to study the evolutionary history of both *AQP2* and *AQP5* genes. The genes were selected from previously obtained reference genomes (see above). When nuclear sequences were obtained, the CDS was translated into protein sequence. Then, protein sequences were aligned with Mafft v.7 (Katoh and Standley 2013). Following Giorgianni et al., (2020), a gene phylogeny was built with the translated CDS sequences, as explained above, using Phyml v.3.3 (Guindon et al. 2010), implementing the Dayhoff substitution model and validating the inferred tree with aBayes support.

### Illumina processing and demographic history

Raw reads for the Illumina data (75 Gb) were filtered, and adapters were removed with fastp v1.0.1 (Chen et al. 2018). A minimum base quality score was set to 30, and adapter detection for paired-end sequencing was activated, with a required fragment length of 50 bp. Trimming of poly-G/X tails and correction in overlapped regions were specified. All other parameters were set as default. Filtered sequences were visually explored with FastQC v0.12.1 (Andrews 2010) to ensure data quality and absence of adapters. Filtered reads were mapped against the new assembly for *T. mauritanica* with bwa-mem v0.7.17 (Li, 2013). Mapped reads were sorted with Samtools v1.9 (Li et al. 2009). Duplicated reads were marked and removed with PicardTools (Broad Institute 2021), and reads with mapping quality lower than 30 were discarded. Then, the demographic history of *T. mauritanica* was inferred using the Pairwise Sequential Markovian Coalescent (PSMC) software v.0.6.5 (Li and Durbin 2011) using the Illumina data. Heterozygous positions were obtained from bam files with the Samtools v.1.9 mpileup function (Li et al. 2009), and data were filtered for low mapping (<30x) and base quality (<30). Minimum and maximum depths were set at a third (6x) and twice (36x) the average coverage. We used the parameter settings -N25 -t15 -r5 -p “1 + 1 + 1 + 1 + 25*2 + 4 + 6” and ran 10 bootstrap replicates to assess the robustness of the inferred demographic trajectories. In addition, we compared this configuration with the original setting -p “4 + 25*2 + 4 + 6” as well as with -p “2 + 2 + 25*2 + 4 + 6”. The squamate mutation rate of 1.1 × 10^−8^ substitutions/site/generation and a generation time of 3.5 years were used, following previous experiences on reptiles (Green et al. 2014; Schield et al. 2022; Burriel-Carranza et al. 2024; Huang et al. 2025; Mochales-Riaño et al. 2025; Talavera et al. 2025), as well as the indications from Hilgers et al. (2025).

## Results and discussion

### Genome assembly

**Fig. 1.**
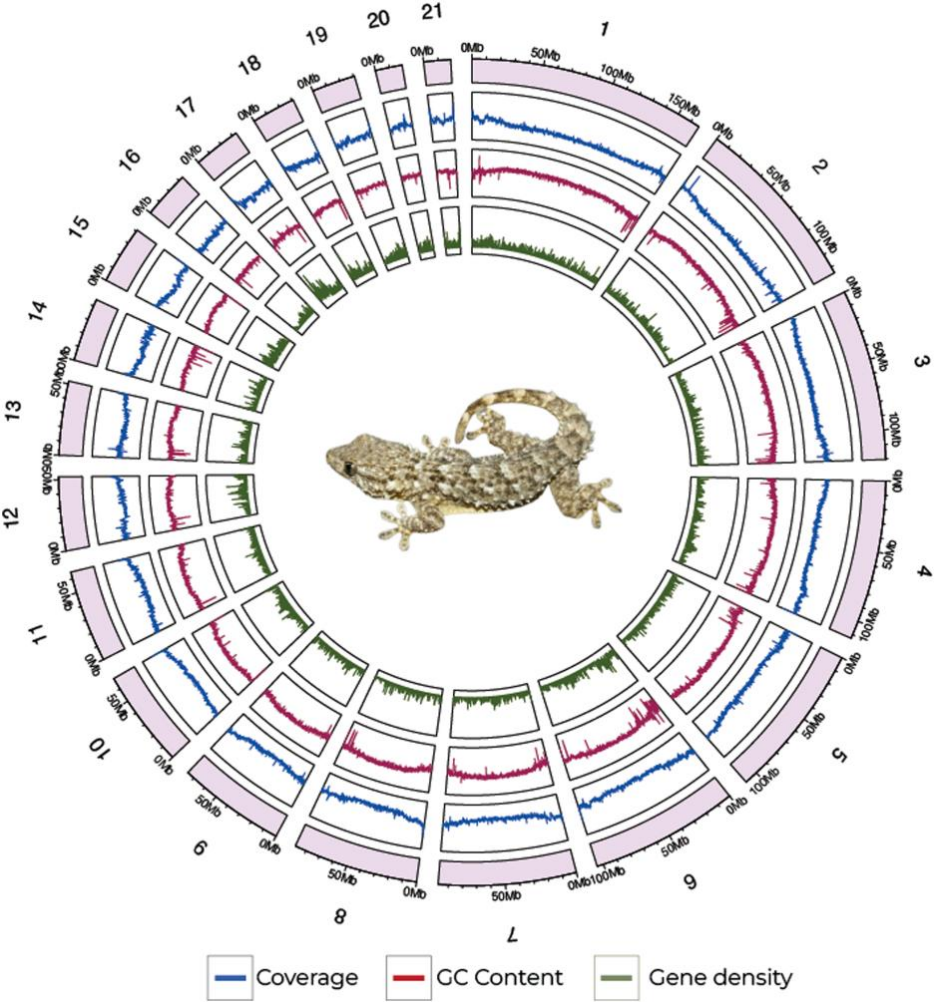
Chromosome-level reference genome for *T. mauritanica*, showing the 21 scaffolds containing more than 96.7% of the genomic information. In blue is shown the coverage levels, in pink the GC content, and in green the gene density along the genome.

We generated a high-quality chromosome-level assembly for the species *T. mauritanica* by combining PacBio HiFi (102.4 Gb of data) and Hi-C (119.6 Gb of data) (Fig. 1, Supplementary Fig. 1 and Table 1). First, the HiFi reads were *de novo* assembled into 702 contigs (N50 = 77 Mb, longest contig 185 Mb). Then, using the linkage data (i.e. Hi-C), the genome was scaffolded into 582 scaffolds (N50 = 135.8 Mb, largest scaffold: 233 Mb), containing 96.7% of the genome in 21 scaffolds or pseudochromosomes (11 macro-, 10 microchromosomes; Fig. 1 and Supplementary Fig. 1). No assembled sex chromosomes were identified based on coverage analysis of Illumina reads, consistent with previous evidence that *T. mauritanica* follows a temperature-dependent sex determination mechanism (Marques et al., 2023; Supplementary Table 1). The total genome length was 2.1 Gb, similar to other gecko species (Burriel-Carranza et al. 2024; Dodge et al. 2025; Pinto et al. 2023; Table 1), and being one of the most contiguous gecko genomes until now (Table 1). BUSCO was used to assess the completeness of the genome using the Sauropsida gene set (*n* = 7,480) (Supplementary Fig. 2). 97.1% of the genes were successfully found (95.9% single-copy, 1.2% duplicated), while the remaining genes were fragmented (0.4%) or missing (2.5%; Supplementary Fig. 2). For the *de novo* assembly, GC content and repeat content were 44.98 and 42.66%, respectively, with the latter being dominated mostly by LINEs (Supplementary Table 2). Finally, a total of 18,847 genes were annotated within the assembly. Functional annotation was assessed by quantifying coverage at the gene level. Functional information, based on Gene Ontology assignments, was provided for 92.68% of the predicted genes (17,469 out of 18,847 total genes), indicating that the annotation set is comprehensive and consistent with expectations for vertebrate genomes of similar size and completeness.

**Table 1.** Comparison of the new reference genome for *T. mauritanica* to other high-quality lizard genomes.

	*T. mauritanica*	*A. caudivolvulus*	*H. binoei*	*E. macularius*	*A. sagrei*
Genome size	2.1 Gb	1.73 Gb	2.6 Gb	2.2 Gb	1.95 Gb
Number of scaffolds	582	37	661	75	**29**
Scaffold N50	135.87 Mb	114.54 Mb	150.61 Mb	145.57 Mb	**249.21 Mb**
Scaffold L50	7	6	7	6	**4**
Contig N50	77 Mb	19.95 Mb	14.37 Mb	**80.1 Mb**	41.14 Mb

Best value per category is shown in bold.

### Chromosome macrosynteny

**Fig. 2.**
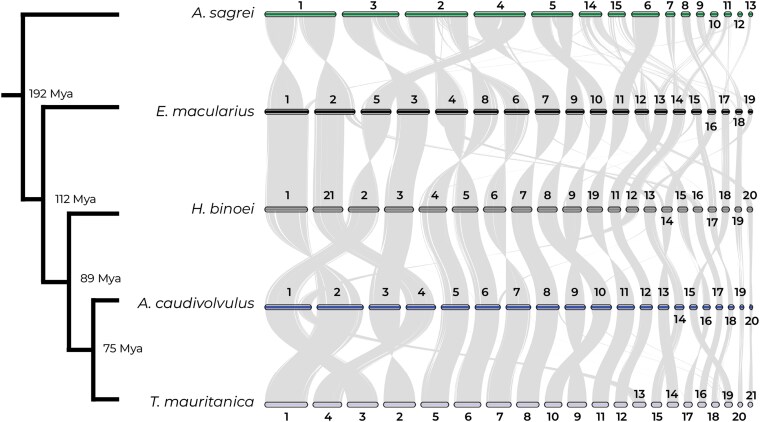
Chromosome-level synteny analyses for the brown Anolis (*Anolis sagrei*), the Leopard gecko (*Eublepharis macularius*), the Bynoe's gecko (*Heteronotia bynoei*), the Emirati leaf-toed gecko (*Asaccus caudivolvulus*), and the Common wall gecko (*Tarentola mauritanica*). Estimates for branch times were obtained from TimeTree.org based on divergence times between species. Please note that apparent full-chromosome inversions are artificial and result from differences in scaffold orientation between assemblies, rather than true structural rearrangements.

Comparative macrosynteny analyses reveal both conserved syntenic blocks and mild chromosomal rearrangements among lizard species (Fig. 2). *Asaccus caudivolvulus* and *T. mauritanica* exhibit a high degree of collinearity, with near one-to-one correspondence of our pseudochromosomes, reflecting their “close” relationship (diverged ∼75 Mya) within the Family Phyllodactylidae (see Gamble et al. 2011) and suggesting relative stability of chromosomal architecture in this family. In contrast, *E. macularius* (Family Eublepharidae) and *H. binoei* (Family Gekkonidae) display more dynamic karyotype evolution, with clear signatures of fissions, fusions, and translocations that have reshaped their genomes. As expected for a more distantly related species (∼192 Mya), the iguanian lizard *A. sagrei* (Family Anolidae) shows the greatest divergence, with widespread rearrangements relative to all gecko species analyzed. These chromosomal patterns indicate that while most large-scale syntenic blocks are conserved across species, some mild chromosomal evolution occurred among their families. The chromosomal stability observed in the Family Phyllodactylidae contrasts with the more dynamic histories inferred for Eublepharidae and Gekkonidae families, suggesting mild lineage-specific trajectories of karyotype evolution. The divergence from *A. sagrei* further highlights the long-term accumulation of rearrangements since the split between geckos and other squamates, emphasizing the role of chromosomal rearrangements in shaping genome organization.

### Aquaporins

**Fig. 3.**
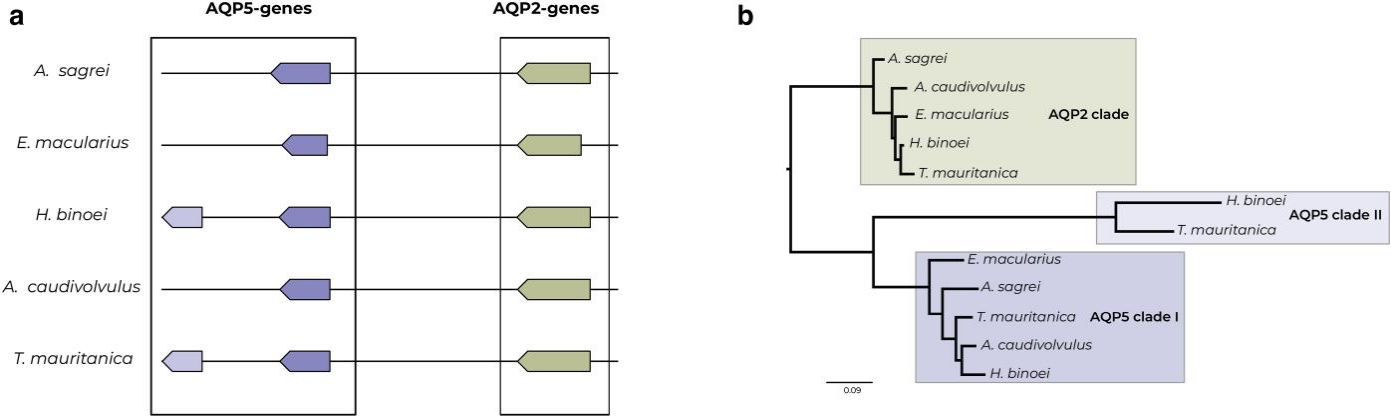
a) Microsinteny analyses for AQP2 and AQP5 genes. Different colors indicate orthologous genes. b) Phylogeny for AQP2 and AQP5 genes, including both genetic clusters identified within the latter. Colored boxes indicate orthology.

The study of the evolution of *AQP* genes revealed that *AQP2* and *AQP5* are generally maintained as single-copy genes in vertebrate genomes, although lineage-specific duplications, such as those observed in the green anole *Anolis carolinensis*, indicate a more dynamic evolutionary history (Finn et al. 2014). Our analyses confirmed that both *AQP2* and *AQP5* genes are typically located on the same chromosome in geckos. However, we discovered a duplication for *AQP5* in both *T. mauritanica* and *H. binoei* (Fig. 3). Phylogenetic reconstructions did not support a recent duplication of this gene in *T. mauritanica*, but rather an older one that was subsequently lost in the phylogenetically closely related *A. caudivolvulus*. Surprisingly, we did not find a duplication for *A. sagrei*, whilst it has been reported for *A. carolinensis* (Finn et al. 2014), highlighting a more complex and lineage-specific evolutionary trajectories of aquaporin genes within squamate taxa (Fig. 3). Functionally, *AQP5* is a water channel with a broad physiological role across vertebrates, including water transport in the salivary glands, trachea, airway epithelia, alveolar cells, and ocular tissues (Krane and Goldstein 2007). In birds, transcriptional downregulation of *AQP5* under osmotic stress has been linked to salt and water balance in the nasal gland (Müller et al. 2006). The presence of an *AQP5* duplication in *T. mauritanica* and *H. binoei* highlights lineage-specific genomic changes within Gekkota. While *AQP5* is functionally associated with water transport across vertebrates (Finn et al. 2014, Martínez-Redondo et al., 2023), our analyses do not establish a direct link between gene duplication and dehydration resistance. Instead, this pattern identifies *AQP5* as a promising candidate for future functional and population-level studies aimed at testing whether copy number variation or regulatory divergence contributes to water balance physiology in arid-adapted geckos. The absence of this duplication in the Emirati leaf-toed gecko *A. caudivolvulus* raises the possibility of lineage-specific gene loss, pointing to an interesting case of genomic flexibility in aquaporin evolution. Although inhabiting the arid United Arab Emirates east coast, previous research on this species suggests its strong relation with relatively high humid habitats (Carranza et al. 2016), which might explain the observed pattern. This finding adds to the growing body of evidence that aquaporin duplications and regulatory changes may have contributed to ecological specialization in reptiles and warrants further investigation through functional approaches. Interestingly, the genus *Tarentola* contains more than 30 described species inhabiting different climatic conditions (Uetz 2025) and differential water loss patterns have already been observed between two different *T. mauritanica* lineages from the Iberian Peninsula (Rato and Carretero 2015). In fact, the specimens collected for this study belong to a mitochondrial clade that has been systematically introduced across the Mediterranean Basin (Rato et al. 2010, 2012, 2016, 2023a, 2023b, 2024) and exhibits greater variability in body water balance in comparison to another lineage of *T. mauritanica* (Rato and Carretero 2015), which is consistent with observed differences in thermal physiology among lineages (Barroso et al., 2020; Mochales-Riaño et al. 2024). Future genomic studies will disentangle whether aquaporin duplications and their regulatory dynamics consistently underlie variation in dehydration resistance across closely related reptile lineages, or whether alternative mechanisms, such as tegumental lipid production and regional differences in skin permeability, play a more prominent role at shallower phylogenetic scales (Weaver et al. 2023; Wu et al. 2025.

### Past demographic history

**Fig. 4.**
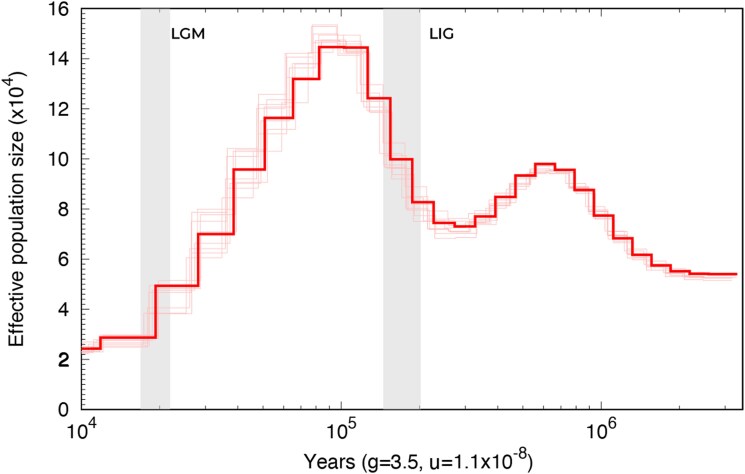
Past demographic reconstruction for *T. mauritanica*. Generation time was set to 3.5 years and the substitution rate to 1.1 × 10-8 per site per year. Last Glacial Maxima (LGM) and Last Interglacial (LIG) are shown with gray lines. The *x*-axis is in millions of years. Red shaded lines represent 10 bootstrap estimates.

PSMC analyses reveal marked fluctuations in the effective population size of *T. mauritanica* over the past one million years (Fig. 4). Population size increased from ∼1 Mya until reaching its maximum between ∼150 and 120 Kya, coinciding with the warmest peak of the Last Interglacial (LIG), which took place around 125 and 129 Kya. After this peak, population size declined significantly, with the most pronounced reduction occurring during the Last Glacial Maximum (LGM, ∼20 Kya). In the postglacial period, population size remained relatively low, with no clear signal of full demographic recovery to pre-LGM levels. The expansion prior to the LIG suggests that favorable interglacial conditions facilitated population growth and range connectivity. In contrast, the decline during the LGM reflects the contraction of suitable habitats caused by colder and drier climates, which restricted populations to Mediterranean refugia. This pattern is consistent with the expectations for Mediterranean species affected by Quaternary climatic oscillations (Araújo et al. 2008). The lack of a strong recovery after the LGM further indicates that postglacial expansions were limited compared to temperate European taxa, possibly due to the adaptation to Mediterranean environments and low colonization events in northern Europe. We did not observe substantial differences when exploring alternative -p parameterizations in PSMC; the inferred timing of demographic contractions and expansions remained largely consistent across configurations (Supplementary Fig. 3). However, the original parameterization produced an apparent recent peak in effective population size, which is likely artefactual, as previously noted by Hilgers et al. (2025).

## Conclusions

Prior to this work, no reference genome was available for the genus *Tarentola*, a diverse clade of geckos adapted to a wide range of environmental conditions. As the first genomic resource for the genus, our assembly provides a solid foundation for comprehensive studies on the evolutionary history of the *Tarentola* genus. Moreover, it will enable investigations into putative differences in gene content associated with ecological adaptations, such as responses to varying humidity regimes. Beyond its value for evolutionary biology, this resource opens opportunities for research across multiple fields. For instance, invasion genomics can be advanced through its application to *T. mauritanica*, a species complex established beyond its native range and capable of thriving under diverse environmental conditions (Rato, et al. 2015; Deso, et al. 2020; Rato, et al. 2021; Silva-Rocha, et al. 2022; Rato, et al. 2024). The results presented here provide a foundation for future ecophysiological studies aimed at linking the genome to phenotype and elucidating why only a single taxon within this species complex has successfully colonized such a wide array of environments. Also, conservation genomic studies can be performed to understand the genetic resilience and vulnerability across the 33 described *Tarentola* species (Uetz 2025). Furthermore, the genome will support studies of physiological and ecological traits of broad relevance to reptiles, including thermal biology, water balance, and osmoregulation. Overall, we hope this reference genome will serve as a fundamental resource for advancing research on gecko biology, from functional genomics to applied studies in conservation and invasion biology, and will stimulate broader comparative genomic analyses across reptiles.

## Supplementary Material

jkag072_Supplementary_Data

## Data Availability

The genome assembly and raw sequencing reads have been deposited in the NCBI under BioProject PRJNA1345349. Raw reads are available through the Sequence Read Archive (SRA) under accessions SRR35879895 (PacBio HiFi), SRR35878998 (Hi-C), SRR35879946 (Illumina), and SRR35889896-SRR35889899 (RNA-seq). All scripts used in the analyses are available upon request. An annotation file for the reference genome assembly is available in a Mendeley Data project accessible at https://data.mendeley.com/datasets/m8hf2v5bxk/1. Supplemental material available at G3 online.
