# Supplementary material for: Chromosome-level reference genome for the common wall gecko (Tarentola mauritanica) enables comparative and functional studies in geckos
Source: G3 (Bethesda). 2026 Mar 31;16(5):jkag072. doi: 10.1093/g3journal/jkag072 (PMC13148402; doi:10.1093/g3journal/jkag072)
Supplement: jkag072_Supplementary_Data [file jkag072_supplementary_data.pdf]

Supplementary figures

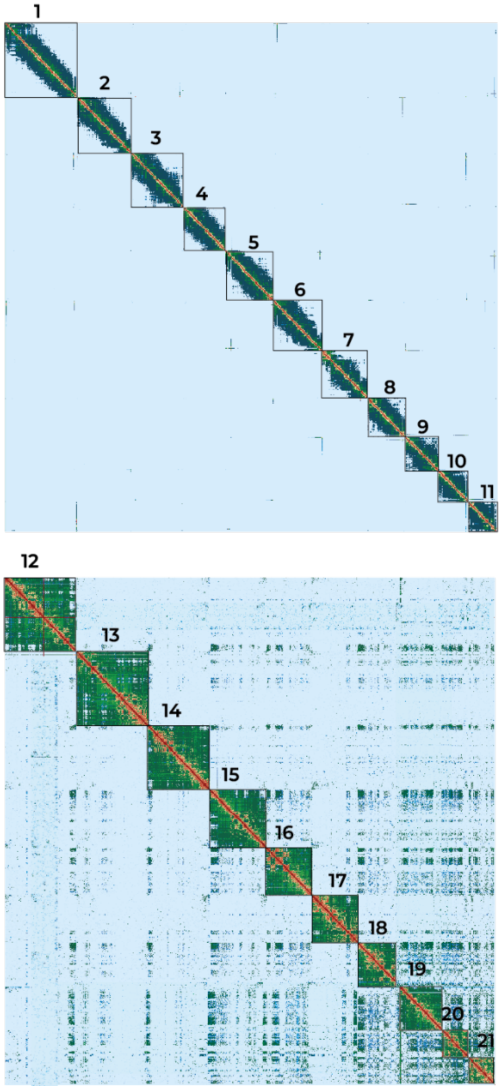

Fig. S1: Contact map showing the 21 pseudochromosomes assembled for the species *T. mauritanica*.

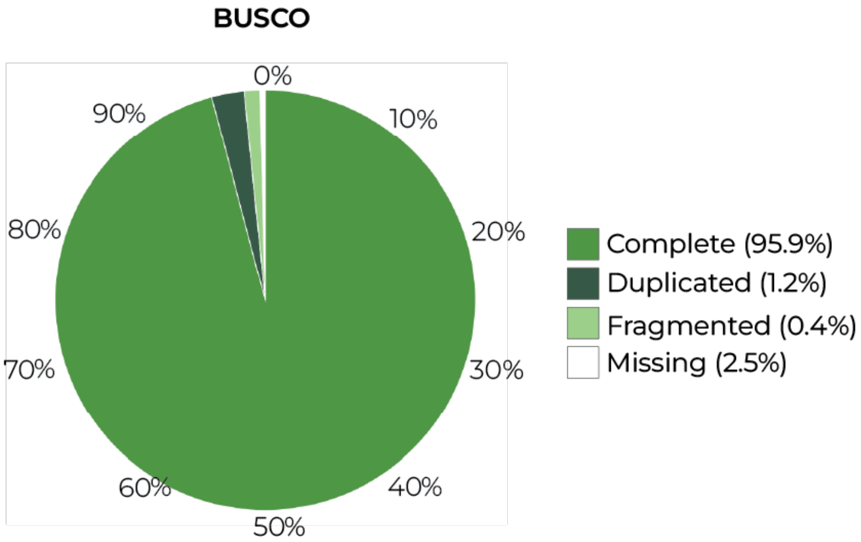

Fig. S2: Busco score against the Sauropsida database (n=7,480) for the new assembled genome for *T. mauritanica*.

A

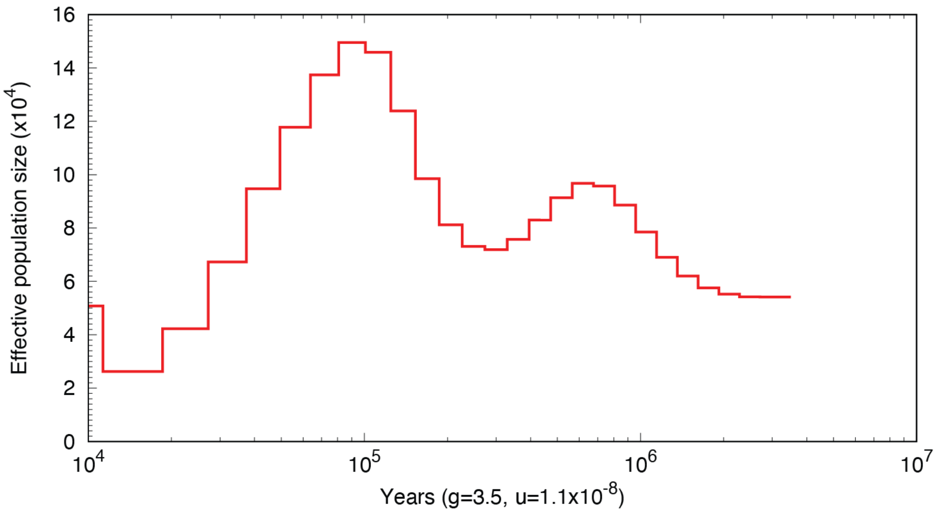

B

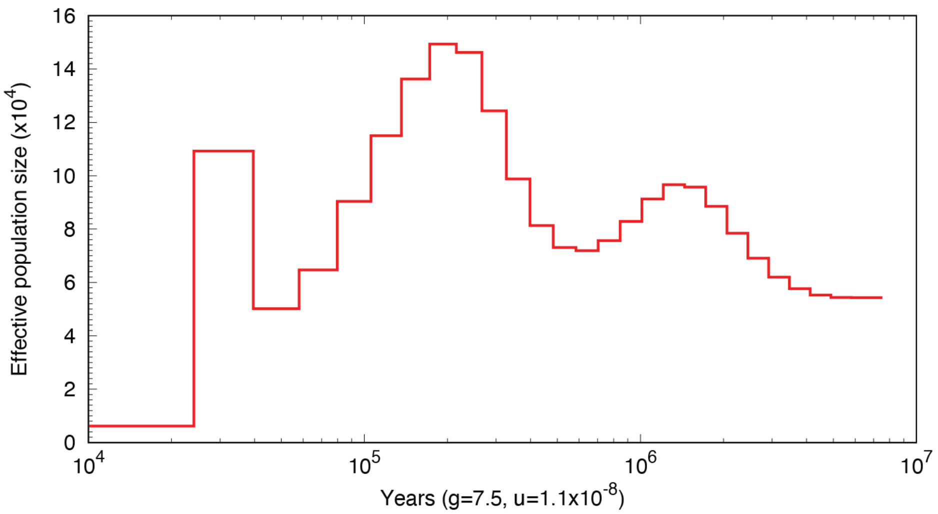

Fig. S3: Demographic analyses using PSMC with different -p parameters (A) -p "2+2+25*2+4+6" and (B) -p "4+25*2+4+6".

Table S1: Mean coverage per chromosome for our newly assembled reference genome for *T. mauritanica*.

Chromosome	Mean coverage
CHR 1	20.47
CHR 2	20.60
CHR 3	20.24
CHR 4	20.33
CHR 5	20.21
CHR 6	20.22
CHR 7	20.24
CHR 8	20.25
CHR 9	20.35
CHR 10	20.34
CHR 11	20.14
CHR 12	19.69
CHR 13	19.42
CHR 14	19.51
CHR 15	19.38
CHR 16	19.88
CHR 17	18.87
CHR 18	19.39
CHR 19	18.72
CHR 20	18.69
CHR 21	18.12

Table S2: Types and proportion of repetitive elements masked within the genome.

Elements	Number of elements	Length	Percentage
SINEs:	863267	82488519 bp	3.82 %
ALUs	1387	48499 bp	0.00 %
MIRs	462199	53372568 bp	2.47 %
LINEs:	2531640	439705724 bp	20.34 %
LINE1	63589	31783517 bp	1.47 %
LINE2	618426	108683222 bp	5.03 %
L3/CR1	1122604	193353150 bp	8.94 %
LTR:	324510	123679912 bp	5.72 %
ERVL	1800	211498 bp	0.01 %
ERVL-MaLRs	305	16516 bp	0.00 %
ERV classI	29423	3495356 bp	0.16 %
ERV classII	6233	898053 bp	0.04 %
DNA elements:	461909	64144224 bp	2.97 %
hAT-Charlie	250295	31350189 bp	1.45 %
TcMar-Tigger	1719	327775 bp	0.02 %
Unclassified	2848721	375487265 bp	17.37 %
Total interspersed repeats		1085505644 bp	50.21 %
Small RNA:	68587	2753948 bp	0.13 %
Satellites:	3580	1439495 bp	0.07 %
Simple repeats:	539558	44066874 bp	2.04 %
Low complexity	59334	2626852 bp	0.12 %
